# Self-Contained Statistical Analysis of Gene Sets

**DOI:** 10.1371/journal.pone.0163918

**Published:** 2016-10-06

**Authors:** David J. Torres, Judy L. Cannon, Ulises M. Ricoy, Christopher Johnson

**Affiliations:** 1 Department of Mathematics and Physical Science, Northern New Mexico College, Española, New Mexico, United States of America; 2 Department of Molecular Genetics and Microbiology, Department of Pathology, University of New Mexico, Health Sciences Center, Albuquerque, New Mexico, United States of America; 3 Department of Biology, Chemistry, and Environmental Science, Northern New Mexico College, Española, New Mexico, United States of America; 4 College of Engineering, Northern New Mexico College, Española, New Mexico, United States of America; National Taiwan University, TAIWAN

## Abstract

Microarrays are a powerful tool for studying differential gene expression. However, lists of many differentially expressed genes are often generated, and unraveling meaningful biological processes from the lists can be challenging. For this reason, investigators have sought to quantify the statistical probability of compiled gene sets rather than individual genes. The gene sets typically are organized around a biological theme or pathway. We compute correlations between different gene set tests and elect to use Fisher’s self-contained method for gene set analysis. We improve Fisher’s differential expression analysis of a gene set by limiting the p-value of an individual gene within the gene set to prevent a small percentage of genes from determining the statistical significance of the entire set. In addition, we also compute dependencies among genes within the set to determine which genes are statistically linked. The method is applied to T-ALL (T-lineage Acute Lymphoblastic Leukemia) to identify differentially expressed gene sets between T-ALL and normal patients and T-ALL and AML (Acute Myeloid Leukemia) patients.

## 1 Introduction

Microarrays allow investigators the opportunity to identify individual genes that are differentially expressed. However, a list of single genes often does not provide insight into different biological themes that distinguish the two phenotypes. For this reason, investigators have sought to incorporate gene sets in their analysis. A priori compiled gene sets group individual genes in biologically related sets. Analyzing gene sets rather than individual genes can improve sensitivity and prediction [1]. For example, a gene set may prove to be significant despite the fact that its individual genes may not be significant [2]. Gene sets can be created based on biological function, metabolic pathway or chromosome. Curated databases include KEGG [3], Reactome [4], Gene Ontology (GO) [5], and the Molecular Signatures Database or MSigDB [6], which serves as a repository for human genes and includes databases from KEGG, Reactome, BioCarta, and GO.

There are two different approaches when analyzing gene sets. The first type (designated *competitive*) compares the gene set with its complement when assessing differential expression. Competitive techniques include the Gene Set Enrichment Analysis (GSEA) [2] and the SAFE technique [7]. The second type (designated *self-contained*) only tests differential expression using the genes within its set.

In the competitive method, the success of a gene set is dependent on the size and nature of its complement. Goeman and Bühlmann [8] advise against the use of competitive methods and Dinu et al. [9] show that GSEA does not properly identify differentially expressed gene sets from a mouse-microarray dataset with simulated genes.

In contrast, self-contained methods, while less popular, only consider those genes within the set for analysis and compute a significance level that is not dependent on genes outside the set. Fridley [10] evaluates a number of self-contained methods. Among them are Stouffer’s method [11], which computes a z-value for the set by “averaging” z-values from the *K* individual genes in the set,

$$\begin{array}{c} Z_{s}=\frac{1}{\sqrt{K}}\sum_{k=1}^{K}Z_{k}, \end{array}$$

and Taylor and Tibshirani (2006) [12], who first order the individual p-values *p*_1_ ≤ *p*_2_ ≤ … ≤ *p*_*K*_ and use the Tail Strength (TS) statistic,

$$\begin{array}{c} TS=\frac{1}{K}\sum_{k=1}^{K}\left[1-p_{k}\left(K+\frac{1}{k}\right)\right]. \end{array}$$

The Kolmogorov-Smirnov (K-S) test [13, 14] computes the maximum difference between two distributions which translates into the statistic,

$$\begin{array}{c} d=max\left\{\frac{k}{K}-p_{k},\frac{k-1}{K}-p_{k}\right\},\;\;1\leq k\leq K. \end{array}$$

Dinu et al. [9] use the *L*_2_ norm of a t-like statistic vector $\sum_{i=1}^{K}d_{i}^{2}$ and a permutation method to assess the significance of a gene set in their Significance Analysis of Microarray to Gene-Set analyses (SAM-GS) method. Others include Tomfohr et al. [15] who use a singular value decomposition of expression levels to identify a metagene which is the eigenvector associated with the largest eigenvalue. Activity levels are compared using the t-test.

Kong et al. [16] use Hotelling’s *T*^2^ statistic (a multiple variable version of the t-test) to assess the significance of a gene set,

$$\begin{array}{c} T^{2}=\frac{n_{1}n_{2}}{n_{1}+n_{2}}{\left.({\bar{X}}_{k}^{\left(1\right)}-{\bar{X}}_{k}^{\left(2\right)}\right)}^{T}S^{-1}\;\left.({\bar{X}}_{k}^{\left(1\right)}-{\bar{X}}_{k}^{\left(2\right)}\right), \end{array}$$

where *n*_1_ and *n*_2_ are the sizes of groups 1 and 2, ${\bar{X}}_{k}^{\left(1\right)}$ and ${\bar{X}}_{k}^{\left(2\right)}$ are the mean vectors of the individual groups, ${\bar{X}}_{k}^{\left(i\right)}=\frac{1}{n_{i}}\sum_{j=1}^{n_{i}}X_{kj}^{\left(i\right)}$, $X_{kj}^{\left(1\right)}$ and $X_{kj}^{\left(2\right)}$ represent the expression level of gene *k* for patient *j* for groups 1 and 2, and **S** is the pooled covariance matrix

$$\begin{array}{c} S=\frac{\left(n_{1}-1\right)S^{\left(1\right)}+\left(n_{2}-1\right)S^{\left(2\right)}}{\left(n_{1}+n_{2}-2\right)} \end{array}$$

where

$$\begin{array}{c} S^{\left(i\right)}=\left\{S_{mp}^{\left(i\right)}\right\}=\frac{1}{n_{i}-1}\sum_{j=1}^{n_{i}}\left(X_{mj}^{\left(i\right)}-{\bar{X}}_{m}^{\left(i\right)}\right)\left(X_{pj}^{\left(i\right)}-{\bar{X}}_{p}^{\left(i\right)}\right) \end{array}$$

is the covariance matrix for group *i*.

Self-contained methods are also used by authors Geoman et al. [17] who base their analysis on a logistic regression model, and Mansmann and Meister [18] who use the Analysis of Covariance (ANCOVA).

We use Fisher’s method [19] which follows a chi-squared distribution to perform our self-contained analysis of gene sets. In addition to having an analytical distribution, Fisher’s method has been shown to be asymptotically Bahadur optimal by Pallini [20]. Fisher’s method, like most self-contained methods, combines the numerical p-values of all the individual genes in the set to form a consolidated p-value. However, caution must be exercised since a few genes can dominate the statistical behavior of the entire gene set. Therefore, we modify Fisher’s method and set a minimum threshold value for individual p-values, thus preventing a few genes from dominating the entire p-value of the gene set. We believe this modification improves the suitability of Fisher’s statistic for evaluating the differential expression of gene sets.

Self-contained methods often employ permutation methods [1] to compute a consolidated p-value since individual genes from gene sets cannot be assumed to be independent. In the permutation approach, the patient expression levels are permuted and the unpermuted test statistic (e.g. Fisher’s F) is evaluated against the statistics (e.g. permuted F values) generated by the permutations. To account for dependencies among individual genes, we also use a permutation method in conjunction with Fisher’s method to evaluate the significance of a gene set.

However, in addition our method evaluates dependencies among pairs of genes during the permutation process and creates a heat map of the dependencies for the gene set. Specifically, we evaluate the probability that gene A is differentially expressed given that gene B is differentially expressed in the arbitrary groups that are created during the permutation process. Thus an investigator not only knows if the gene set is significant but what genes are linked together within the set. A high level of dependency among the genes in a gene set may increase the set’s potential to be selected as a differentially expressed set.

We apply the method to identify differentially expressed gene sets when T-ALL (T-lineage Acute Lymphoblastic Leukemia) patients are compared to healthy patients and AML (Acute Myeloid Leukemia) patients using Affymetrix microarray datasets. We use the publicly available Gene Expression Omnibus database (http://www.ncbi.nlm.nih.gov/geo/) accession numbers GSE46170 [21], GSE13204 [22–24], and GSE36133 [25] for our analysis. Microarray chip Human Genome U133 Plus 2.0 was used in the databases. Preprocessing and normalization for GSE13204 and GSE36133 are discussed in [23, 26] and [25] respectively.

Our paper is organized as follows. We discuss Fisher’s method and our modification to Fisher’s method in Section 2. Fisher’s method is also compared to other self-contained methods using a correlation and power study. Section 3 describes how dependencies in a gene set are accounted for and computed. When many gene sets are tested for significance, there is an increased probability that one may find false positives. We adjust for the multiple tests through the false discovery rate which is discussed in Section 4. Section 5 discusses our results using the Gene Expression Omnibus datasets and Section 6 concludes.

## 2 Analyzing gene sets for differential expression using Fisher’s method

Given the probability levels *p*_*k*_ of *K* individual genes, Fisher [19] combines the p-values in a set using the expression,

$$\begin{array}{c} F=-2\sum_{k=1}^{K}ln\left(p_{k}\right)=-2ln\left(\prod_{k=1}^{K}p_{k}\right). \end{array}$$ (1)

When the individual genes are independent, *F* follows a chi-squared distribution with 2*K* degrees of freedom from which a consolidated p-value can be determined for the entire set.

Table 1 compares pairs of self-contained methods by constructing Pearson’s *r* coefficients. Each entry in the table computes *r* from 100,000 p-values from two different self-contained methods. The p-values are themselves computed using two simulated gene sets, each composed of *K* = 20 genes and *n* = 100 patients generated by sampling from a standard normal distribution (*μ* = 0, *σ* = 1). We see that Fisher’s method is highly correlated with SAM-GS and Stouffer’s method.

**Table 1:** Correlation of Fisher’s method with other self-contained methods for gene set analysis.

	Fisher	SAM-GS	Stouffer	Hotelling *T*^2^	TS	K-S
Fisher	1.0	.99	.98	.88	.87	.77
SAM-GS	.99	1.0	.94	.89	.78	.70
Stouffer	.98	.94	1.0	.83	.95	.85
Hotelling *T*^2^	.88	.89	.83	1.0	.70	.62
TS	.87	.78	.95	.70	1.0	.90
K-S	.77	.70	.85	.62	.90	1.0

One vulnerability of Fisher’s method (and other self-contained methods) is that a small subset of genes can conspire to generate a small consolidated p-value for the entire set of K genes. Whitlock [27] notes that Fisher’s method is asymmetrically sensitive to small p-values and elects to used a weighted Z-method. Table 2 shows the average p-values of varying gene subsets that will cause the entire set to be significant at a probability level of *α* = .01. For example, a single gene whose p-value is 3.5 × 10^−6^ or less will cause the entire set of 10 genes to be significant at a consolidated p-value of *α* = .01. We assume the remaining 9 genes (or K-1 genes in general) to have p-values of.5. Similarly, three genes whose p-values are 1.2 × 10^−3^ or less will cause the entire set of 20 genes to be significant.

**Table 2:** Computed p-values of highest ranked genes required to make the entire set of *K* genes significant at *α* = .01 using Fisher’s method.

K	p (1 gene)	p (2 genes)	p (3 genes)	p (4 genes)	p (5 genes)
10	3.5 × 10^−6^	1.3 × 10^−3^	9.6 × 10^−3^	2.6 × 10^−2^	4.7 × 10^−2^
20	7.7 × 10^−9^	6.2 × 10^−5^	1.2 × 10^−3^	5.6 × 10^−3^	1.4 × 10^−2^
40	2.3 × 10^−13^	3.4 × 10^−7^	3.8 × 10^−5^	4.1 × 10^−4^	1.7 × 10^−3^
80	2.4 × 10^−21^	3.4 × 10^−11^	8.4 × 10^−8^	4.1 × 10^−6^	4.3 × 10^−5^

To prevent a few genes from dominating the statistical significance of the entire gene set, we modify Fisher’s method. Our adjusted Fisher’s test sets a lower limit *p*_*min*_ on p-values

$$\begin{array}{c} F=-2\sum_{k=1}^{K}ln\left(max\left\{p_{k},p_{min}\right\}\right), \end{array}$$ (2)

where *p*_*min*_ is a small parameter, say 10^−2^. Zaykin et al. (2002) [28] also modify Fisher’s method, but in contrast, limit the maximum value for p-values to improve the statistical power for rejecting a null hypothesis. Concern for false positives motivates Chai et al. [29] to adjust Fisher’s method using Brown’s approximation [30].

**Fig 1:**
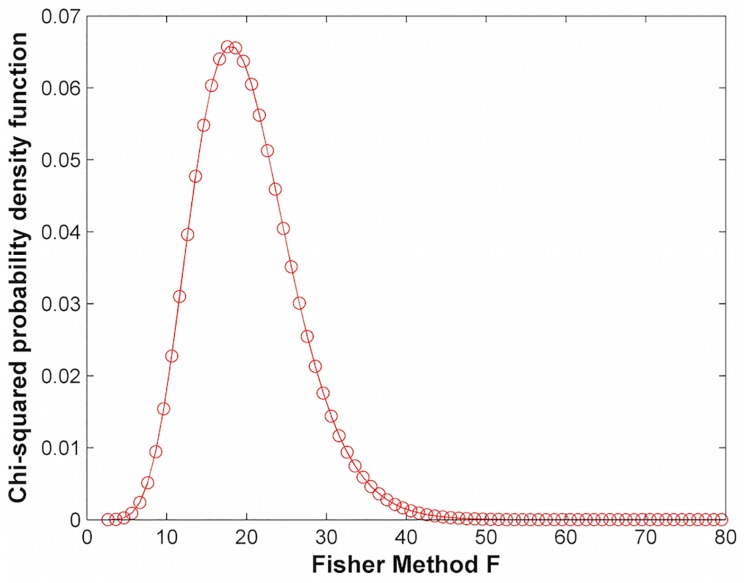
Chi-squared distribution corresponding to K = 10 (20 degrees of freedom).

**Fig 2:**
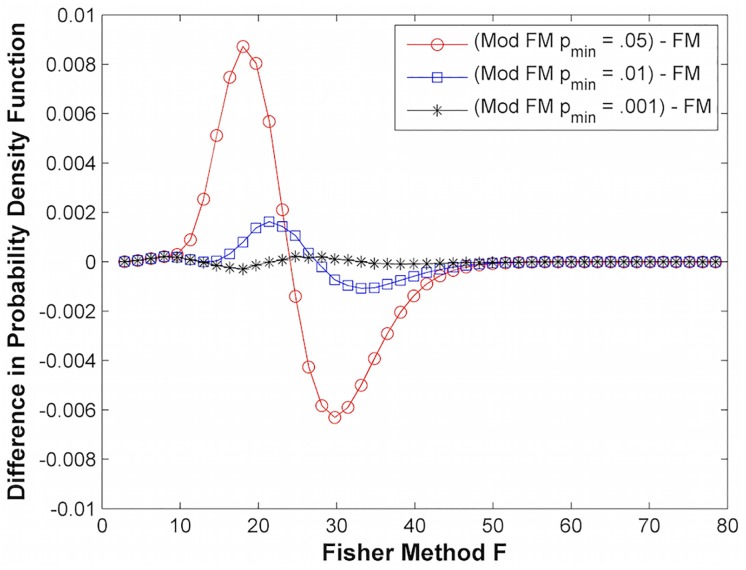
Difference in probability density functions (Modified Fisher’s method (Mod FM) and Fisher’s method (FM)) at different minimum p-values.

The probability density function (PDF) of our modified Fisher’s method can be constructed for different values of *p*_*min*_ in order to properly evaluate the p-value associated with an *F* value. Fig 1 plots the chi-squared distribution with 20 degrees of freedom (*K* = 10 genes) and Fig 2 shows the difference between the PDFs of the modified Fisher’s method (Mod FM) and Fisher’s method (FM) generated by sampling p-values from a uniform distribution of *K* = 10 genes. As expected, the difference between the PDF of the modified Fisher’s method and Fisher’s method decreases as *p*_*min*_ decreases.

**Fig 3:**
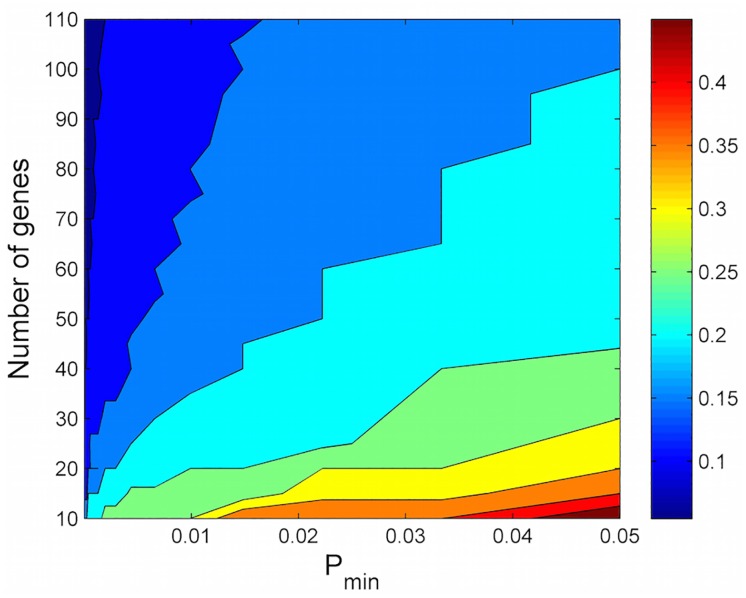
Proportion of genes required to achieve a global gene set significance of *α* = .01 using the modified Fisher’s method at different levels of *p*_*min*_ and different gene set sizes.

The PDF of the modified Fisher’s method can then be used to determine the minimum number of genes required to make the consolidated p-value of the gene set less than some value *α*. Specifically, we determine the smallest value *K*_*min*_ such that

$$\begin{array}{c} -2\left(\sum_{k=1}^{K_{min}}ln\left(p_{min}\right)+\sum_{k=K_{min}+1}^{K}ln\left(.5\right)\right)>F_{\alpha } \end{array}$$ (3)

where *F*_*α*_ is the value of *F* for which the area to the right of the PDF of the modified Fisher’s method is less than *α*. Table 3 shows the minimum number of genes required to achieve a gene set significance level of *α* = .01 for the modified Fisher’s method using different levels of *p*_*min*_ and different gene set sizes. As *p*_*min*_ increases, more genes need to have individual p-values of *p*_*min*_ or less. For the unmodified Fisher’s method (*p*_*min*_ = 0) or chi-squared distribution, only one gene is required. We also note that the proportion of genes required to have p-values of *p*_*min*_ or less decreases as the gene set size increases. Fig 3 shows the proportion of genes that need to have individual p-values of *p*_*min*_ or less in order for the entire gene set to achieve a significance level of *α* = .01 for different gene set sizes and levels of *p*_*min*_.

**Table 3:** Minimum number of genes required to achieve a global gene set significance of *α* = .01 using the modified Fisher’s method at different levels of *p*_*min*_.

Number of genes (K)	*p*_*min*_ = .05	*p*_*min*_ = .01	*p*_*min*_ = .001	*p*_*min*_ = 0
10	5	3	2	1
20	7	5	3	1
40	11	7	5	1
80	17	12	8	1

One potential concern in using the modified Fisher’s method is that the probability density function (PDF) for different combinations of *p*_*min*_ and gene set sizes *K* needs to be constructed. However, in our gene set analysis, we avoid having to compute the PDF because we use the permutation method (as discussed in the next section) to evaluate the p-value of a gene set which is based on a ranking of Fisher’s *F* values. Thus the need to extract the p-value associated with an *F* value from the modified probability density function is eliminated.

To further evaluate the modified Fisher’s method, we compare the power of each self-contained method in Table 4 by creating two gene sets each composed of *K* = 20 genes and *n* = 50, *n* = 100, or *n* = 200 patients. Expressions levels are created by sampling from a standard normal distribution (*σ* = 1) with a mean of (*μ* = 0) for the first set and a mean of (*μ* = .15) for the second set. Power is determined using 10,000 permutations. As expected, the power increases as the number of patients increases. Fisher’s method exhibits equal or slightly higher power compared to other methods and shows little variation in power as *p*_*min*_ changes. The higher power of Fisher’s method is consistent with the results from Fridley et al. [10]. Table 5 compares the fraction of incorrect *H*_0_ rejections (Type I errors) for different self-contained methods. Two gene sets are created, each of which is composed of *K* = 20 genes and *n* = 100 patients. Expression levels are created for each set by sampling from a standard normal distribution (*μ* = 0, *σ* = 1). Table 5 shows the fraction of the 10,000 genes sets which produce p-values (evaluated using 10,000 permutations) that are less than.05. We see that most methods commit approximately.05 Type I errors. The value of *p*_*min*_ has little effect on the fraction of Type I errors committed by Fisher’s method.

**Table 4:** Power of different self-contained gene set methods for different patient sizes, *K* = 20 genes, and 10,000 gene sets. Expressions levels are created by sampling from a standard normal distribution (*σ* = 1) with a mean of (*μ* = 0) for the first set and a mean of (*μ* = .15) for the second set.

Number of Patients	Fisher *p*_*min*_ = 0	Fisher *p*_*min*_.001	Fisher *p*_*min*_.01	SAM-GS	Stouffer	*T*^2^	TS	K-S
50	.45	.45	.44	.44	.44	.37	.36	.32
100	.84	.84	.83	.83	.83	.79	.74	.68
200	.997	.996	.996	.996	.996	.994	.986	.977

**Table 5:** Fraction of Type I errors for gene set methods, K = 20 genes, n = 100 patients, 10,000 gene sets. Expressions levels for each gene set are created by sampling from a standard normal distribution (*μ* = 0, *σ* = 1).

	Fisher *p*_*min*_ = 0	Fisher *p*_*min*_ = .1, (.01)	SAM-GS	Stouffer	*T*^2^	TS	K-S
Fraction of Type I errors	.049	.05, (.049)	.051	.049	.051	.049	.048

## 3 Accounting for dependencies among genes

Fisher’s method assumes the genes in a gene set act independently. However genes in a set are often grouped together because they share a common biological function. Thus gene independence cannot be assumed. To overcome this dilemma, investigators (e.g. [8], [9], [10], [16]) and we compute a distribution by permuting the patient phenotypic labels. The p-value of the gene set is then determined by comparing the rank of the unpermuted statistic relative to the other permutations.

**Fig 4:**
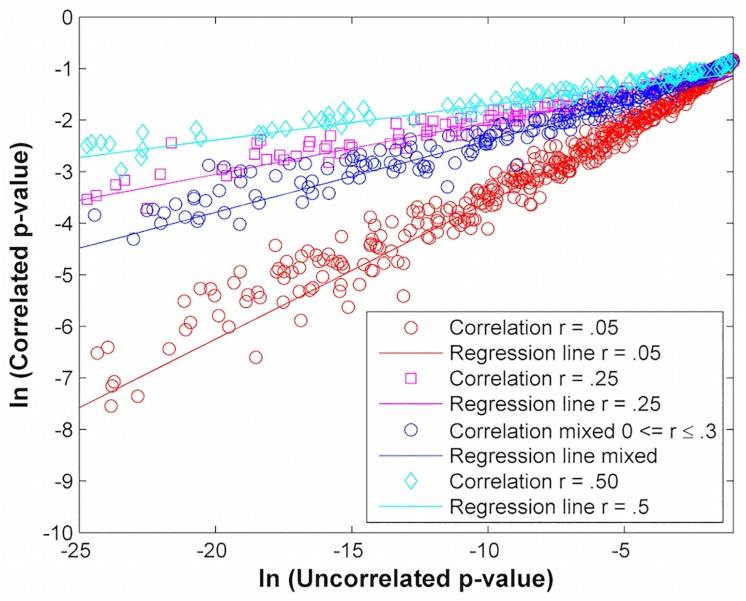
*ln*(*p*_*corr*_) vs *ln*(*p*_*uncorr*_) at different correlation levels (r) where *p*_*corr*_ represents the correlated p-value and *p*_*uncorr*_ represents the uncorrelated p-value.

To assess the difference in p-values for correlated and independent genes, we perform a simulation where 100 gene expression levels of 200 patients are randomly sampled from two normal distributions (100 patients in each group) for different levels of correlation *r*. The p-value of the gene set is computed with Fisher’s method (which assumes the genes are independent and uncorrelated) and with 100,000 permutations (which accounts for the fact that the genes are correlated). Let us denote the former by *p*_*uncorr*_ and the latter by *p*_*corr*_. We compute *p*_*uncorr*_ by using the cumulative chi-squared distribution and *p*_*corr*_ by ranking the Fisher’s *F* values. For the purposes of this numerical experiment, our simulation uses the unmodified Fisher’s method, *p*_*min*_ = 0. The genes are correlated by constructing and Cholesky factoring a correlation matrix for different levels of correlation *r*. Subsequently, the *ln*(*p*_*uncorr*_) values are plotted on the x-axis and the *ln*(*p*_*corr*_) values are plotted on the y-axis at different correlation levels. A linear regression line is then fitted for each correlation level *r*, *r* = .05, *r* = .25, *r* = .50 and mixed correlation levels (*r* = .15 + .15*sin*(2*πk*_1_ *k*_2_) where *k*_1_ and *k*_2_ refer to two different genes). We use the equation

$$\begin{array}{c} p_{corr}=bp_{uncorr}^{m} \end{array}$$ (4)

or equivalently

$$\begin{array}{c} ln\left(p_{corr}\right)=m\;ln\left(p_{uncorr}\right)+ln\left(b\right) \end{array}$$ (5)

to model the relationship between *p*_*corr*_ and *p*_*uncorr*_. Eq (5) can be used to fit a least squares regression line through the data. For example, for correlation level *r* = .05, we calculate *m* = .27 and *b* = .40. Fig 4 shows the relationship between the correlated p-value and the uncorrelated p-value. We notice three trends. First, as expected, *p*_*corr*_ is higher than *p*_*uncorr*_ for *r* > 0. Second, the ratio *p*_*corr*_/*p*_*uncorr*_ increases as the level of correlation (*r*) increases, which is also not surprising. Finally, the ratio *p*_*corr*_/*p*_*uncorr*_ increases as *p*_*uncorr*_ decreases. Table 6 further illustrates this third trend by comparing *p*_*uncorr*_, *p*_*corr*_, and the ratio *p*_*corr*_/*p*_*uncorr*_ at correlation level *r* = .05 and calculated values *m* = .27 and *b* = .40. We see that the ratio *p*_*corr*_/*p*_*uncorr*_ is only 12 for *p*_*uncorr*_ = 10^−2^ but 2.5 × 10^8^ for *p*_*uncorr*_ = 10^−12^. The benefit of fitting a regression line using Eq (5) is that *m*, the slope of the line, and *b*, its y-intercept, can be used in Eq (4) to predict the correlated p-value (*p*_*corr*_) using its uncorrelated (*p*_*uncorr*_) value. We find that for the databases encountered in Section 5.2, such an approach is required since the number of permutations needed to compute *p*_*corr*_ is prohibitively large. The coefficients *m* and *b* in Eq (5) are first extracted from the regression line using a computationally acceptable number of permutations. Then the permutated p-value or *p*_*corr*_ is extrapolated using Eq (4). Nonzero values of *p*_*min*_ can also be accommodated since the functional relationship between *p*_*corr*_ and *p*_*uncorr*_ is built during the permutation process.

**Table 6:** Relationship between *p*_*uncorr*_ (uncorrelated p-values) and *p*_*corr*_ (correlated p-values) at correlation level *r* = .05.

*p*_*uncorr*_	10^−2^	10^−4^	10^−6^	10^−8^	10^−10^	10^−12^
*p*_*corr*_	.12	.034	.01	.003	.00087	.00025
*p*_*corr*_/*p*_*uncorr*_	12	340	1.0 × 10^4^	3.0 × 10^5^	8.7 × 10^6^	2.5 × 10^8^

We also compute dependencies between each pair of genes in the gene set during the permutation process. To accomplish this, we compute the proportion of permutations in which gene A is significant (at some p-value level), gene B is significant, and genes A and B are simultaneously significant. These proportions correspond to the probabilities *P*(*A*), *P*(*B*) and *P*(*AandB*). Since *P*(*AandB*) = *P*(*A*)*P*(*B*|*A*) and *P*(*AandB*) = *P*(*B*)*P*(*A*|*B*), the probabilities *P*(*B*|*A*) and *P*(*A*|*B*) can be computed. A heat map of *P*(*A*|*B*)/*P*(*A*) for all pairs of genes A and B in a gene set can be plotted. If *P*(*A*|*B*)/*P*(*A*) > 1, the differential expression of *P*(*B*) increases the probability that gene A is differentially expressed by factor *P*(*A*|*B*)/*P*(*A*). We also note that the factor *P*(*A*|*B*)/*P*(*A*) is symmetric, *P*(*A*|*B*)/*P*(*A*) = *P*(*B*|*A*)/*P*(*B*). We emphasize for clarity that, due to the arbitrary groups created during the permutation process, *P*(*A*) and *P*(*B*) do not represent the probability that genes A and B are differentially expressed for the original unpermutated groups.

## 4 False discovery rate

Since many gene sets are tested in our method, we must account for the increased probability of achieving false positives when using multiple tests. We choose not to use the Family Wise Error Rate (FWER) which reduces the probability that one or more false positives are reported to be less than *α* since FWER methods suffer from increased Type II errors [31]. Instead, we use the false discovery rate (FDR) method of Benjamini and Hochberg [32]. The false discovery rate is the expected fraction of false positives in the number of reported positives.

In the Benjamini and Hockberg (BH) method [32], the p-values of all sets are ordered from smallest to largest. Then the largest index *k* is found such that $p_{k}<\frac{k\alpha }{K}$. All alternative hypothesis are retained for gene sets *i* ≤ *k*.

## 5 Applying the method to T-ALL

Pediatric acute lymphoblastic leukemia (ALL) is the most common childhood cancer and the T-lineage subtype (T-ALL) has a poorer prognosis than B-lineage [33]. Many microarray analyses of T-ALL disease have been done, leading to identification of genes that are involved with the disease [34]. Whole-genome sequencing of twelve early T-cell precursor acute lymphoblastic leukemia (ETP ALL) patients revealed mutations in histone-modifying genes, genes related to cytokine receptor and RAS signalling, and lesions involving haematopoietic development [34]. Despite advances in treatment leading to a high cure rate, there are still significant therapeutic barriers in treating relapse disease [33]. Thus, there is interest in identify genetic signatures of T-ALL relapse disease.

In addition, acute lymphoblastic leukemia is a disease where the success of treatment is linked to identifying the leukemia subtype and tailoring the treatment to the subtype [35]. Yeoh et al. [35] identify clusters of genes by using expression profiles of the the top genes for each subgroup. T-ALL is distinguished from other acute lymphoblastic leukemias by the CD3D gene. Subgoups of T-ALL can be determined through the stage of T-cell development and the T-ALL oncogenes: HOX11L2, LYL1 plus LMO2, TAL1 plus LMO1 or LMO2, HOX11, and MLL-ENL (Ferrando and Look [36], Pui et al. [37]). Classification can help determine prognosis based on treatment regimes. For example, MLL-ENL [37] and HOX11 (when treated with combination chemotherapy [36]) have favorable prognoses. HOX11L2 was shown to be a subtype of pediatric T-ALL with poor prognosis (Ballerini et al. [38]). Ferrando et al. [39] link T-ALL genes HOX11, TAL1, and LYL with immunophenotypic expression and stages of thymocyte differentiation. The less favorable prognosis of TAL1 and LYL1 subtypes could be attributed to upregulation of antiapoptotic genes (BCL2A1 or BCL2) [39]. According to Pui et al. [37], “many novel genomic alterations have recently been identified, including focal deletions leading to dysregulated expression of TAL1 and LMO2, deletion and mutation of PTEN, mutations of NOTCH1 and FBXW7, deletions of RB1, duplication of MYB, deletions of RB1, and fusion of SET or ABL1 to NUP214,” confirming that T-ALL is a heterogeneous disease. However, unraveling which genes are the drivers and which are passengers in gene expression analysis can be a challenge [37].

Maiorov et al. [40] use a network-based classification scheme and compare T-ALL patients with normal patients using Gene Expression Omnibus databases GSE13204 and GSE46170. They identify 19 significant subnetworks containing 102 genes and conclude that, “transcription factors, zinc-ion-binding proteins, and tyrosine kinases are the important protein families to trigger T-ALL.” Maiorov et al. assemble the following genes {1. ABL1, 2. CCL5, 3. CD99, 4. TP53, 5. WT1} which have been linked with T-ALL from associated studies and which have been identified in their subnetworks. We calculate the p-values of these genes using the t-test and database GSE13204 to be respectively 3.9 × 10^−31^, 3.0 × 10^−34^, 2.8 × 10^−66^, 1.1 × 10^−8^, and 3.1 × 10^−22^ which confirms their significance.

### 5.1 Gene Expression Omnibus Accession Number GSE46170

Using our modified Fisher’s method with *p*_*min*_ = .001, the Gene Expression Omnibus dataset GSE46170, and the false discovery rate of.0025, we identify the following significant gene sets shown in Table 7 from BioCarta, KEGG, Reactome, and Hallmark which have been downloaded from the MSigDB database [2]. We link only one probe with each gene. The caption of Table 7 includes a description of each gene set from MSigDB [2]. According to [21], “RNA was isolated from the bone marrow samples of childhood T-ALL patients at the time of diagnosis with a blast count over 90% and hybridized to Affymetrix GeneChip HU-133 Plus.2.” Table 7 lists the database associated with each gene set, the number of genes in the set, and the consolidated p-value of each set. The number in parentheses is the number of genes actually found in GSE46170. For each individual gene, 31 T-ALL patients and 7 healthy patients were used to compute a p-value based on the Wilcoxon rank-sum test. A permutation method with 100,000 permutations was used to generate the consolidated p-value of the gene set. (Incidentally, Stouffer’s method also identified genes sets tagged with an asterisk (*) using a false discovery rate of.0025.)

**Table 7:** Gene sets and associated p-values that are differentially expressed (T-ALL versus Healthy) using Gene Expression Omnibus Accession GSE46170 and a False Discovery Rate of.0025. Individual genes within each set can be found at software.broadinstitute.org/gsea/msigdb [2]. Individual gene p-values are computed with the Wilcoxon rank-sum test. Gene sets identified with an asterisk (*) were also identified by Stouffer’s method. **Description of gene sets in Table 7** taken from Subramanian et al. [2]. 1. “Deregulation of CDK5 in Alzheimers Disease” 2. “Genes involved in Pre-NOTCH Transcription and Translation” 3. “Genes involved in Regulation of Complement cascade” 4. “Genes involved in p38MAPK events” 5. “Oxidative Stress Induced Gene Expression Via Nrf2” 6. “Genes involved in Signaling by BMP” 7. “Genes involved in Elevation of cytosolic Ca2+ levels” 8. “Genes up-regulated during formation of blood vessels (angiogenesis)” 9. “Genes involved in Synthesis, Secretion, and Inactivation of Glucose-dependent Insulinotropic Polypeptide (GIP)”.

	GENE SET	DATABASE	Number of genes	p-value
1	(*)BIOCARTA_ P35ALZHEIMERS_PATHWAY	Biocarta	11 (11)	1 × 10^−6^
2	(*)REACTOME_PRE_NOTCH_ TRANSCRIPTION_AND_TRANSLATION	Reactome	29 (27)	1 × 10^−6^
3	(*)REACTOME_REGULATION_ OF_COMPLEMENT_CASCADE	Reactome	14 (13)	1 × 10^−6^
4	(*) REACTOME_P38MAPK_EVENTS	Reactome	13 (13)	1 × 10^−6^
5	BIOCARTA_ARENRF2_PATHWAY	Biocarta	13 (13)	2 × 10^−6^
6	REACTOME_SIGNALING_BY_BMP	Reactome	23 (22)	2 × 10^−6^
7	(*)REACTOME_ELEVATION_ OF_CYTOSOLIC_CA2_LEVELS	Reactome	10 (8)	2 × 10^−6^
8	HALLMARK_ANGIOGENESIS	Hallmark	36 (36)	2 × 10^−6^
9	(*)REACTOME_SYNTHESIS_ SECRETION_AND_INACTIVATION_OF_GIP	Reactome	14 (12)	2.1 × 10^−5^

We focus our attention on one of the gene sets in Table 7 (REACTOME_PRE_NOTCH_TRANSCRIPTION_AND_TRANSLATION) whose 29 individual genes are: 1. MAMLD1, 2. CREBBP, 3. E2F1, 4. E2F3, 5. EIF2C3, 6. EIF2C4, 7. EP300, 8. SNW1, 9. TNRC6B, 10. KAT2A, 11. EIF2C1, 12. EIF2C2, 13. TNRC6A, 14. RBPJ, 15. JUN, 16. MOV10, 17. LOC441488, 18. NOTCH2, 19. NOTCH3, 20. NOTCH4, 21. MAML3, 22. TNRC6C, 23. CCND1, 24. TFDP1, 25. TP53, 26. LOC728030, 27. MAML2, 28. KAT2B, and 29. MAML1. Descriptions of these individual genes can be found at http://software.broadinstitute.org/gsea/msigdb. Genes LOC441488 and LOC728030 are the only genes out of the 29 that were not located in the GSE46170 dataset and not included in the gene set analysis. The gene set, REACTOME_PRE_NOTCH_TRANSCRIPTION_AND_TRANSLATION, compiles genes involved in “Pre-Notch transcription and translation” [2]. NOTCH1 is a transcription factor involved in “multiple stages of T-cell development” [41]. Mutations in NOTCH1 have been found in over 50% of T-ALL cases [41].

**Fig 5:**
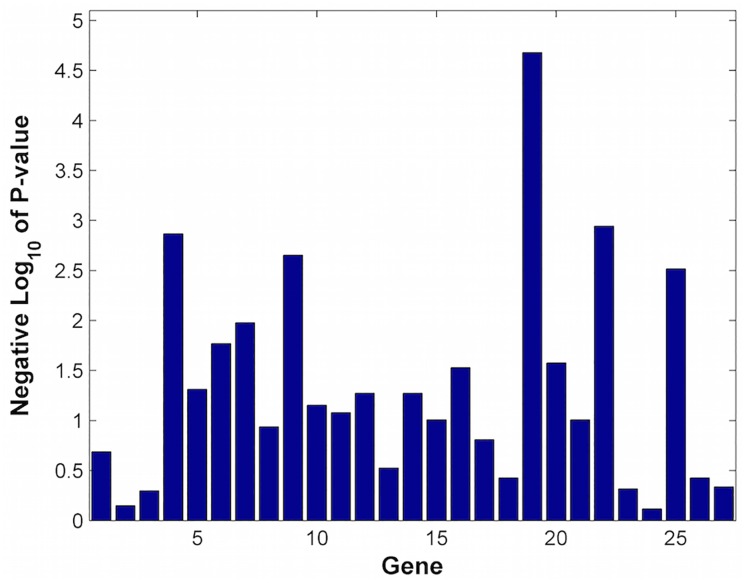
−*log*_10_(*p*_*value*_) of genes in REACTOME_PRE_NOTCH_TRANSCRIPTION_AND_TRANSLATION. The Wilcoxon rank-sum test is used to compute the p-values of each gene.

**Fig 6:**
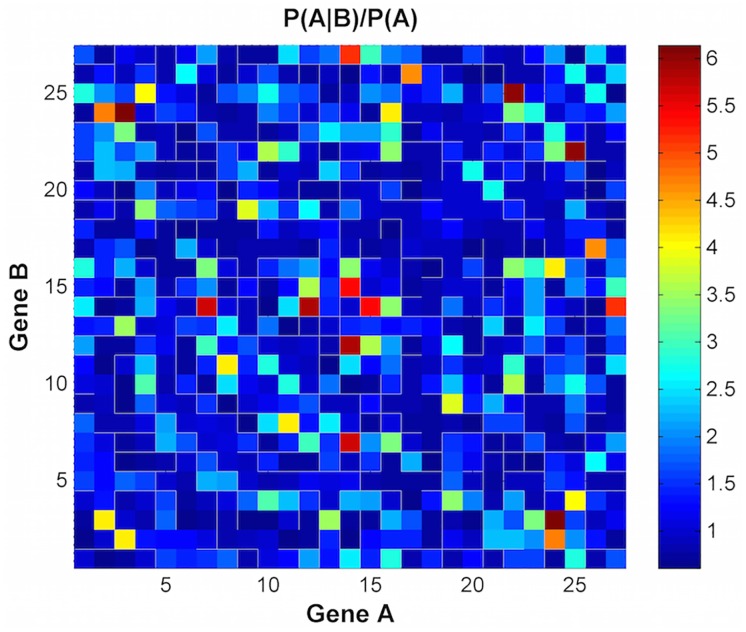
The multiplicative factor P(A | B)/P(A) is the increased probability that gene A is differentially expressed (at p-value = .05 or less) given the differential expression of gene B (at p-value = .05 or less) for REACTOME_PRE_NOTCH_TRANSCRIPTION_AND_TRANSLATION.

Fig 5 plots the −*log*_10_ of the p-values of the genes in the set using a Wilcoxon rank-sum test. We see that gene 4. E2F3, 9. TNRC6B, 19. NOTCH3, 22. TNRC6C, and 25. TP53 are highly significant. Fig 6 plots P(A | B)/P(A) to show the dependencies among the genes. The multiplicative factor P(A | B)/P(A) is the increased probability that gene A is differentially expressed (at p-value.05 or less) given the differential expression of gene B (at p-value.05 or less). For example, the dark red squares are genes whose probability of being differentially expressed is 5–6 times higher if the gene it is paired with is differentially expressed. We see that the following gene pairs have a positive dependence: gene 3 (E2F1) and gene 24 (TFDP1); gene 7 (EP300) and gene 14 (RBPJ); gene 12 (EIF2C2) and gene 14 (RBPJ); and gene 22 (TNRC6C) and gene 25 (TP53).

### 5.2 Challenges posed by microarrays GSE13204 and GSE36133

**Fig 7:**
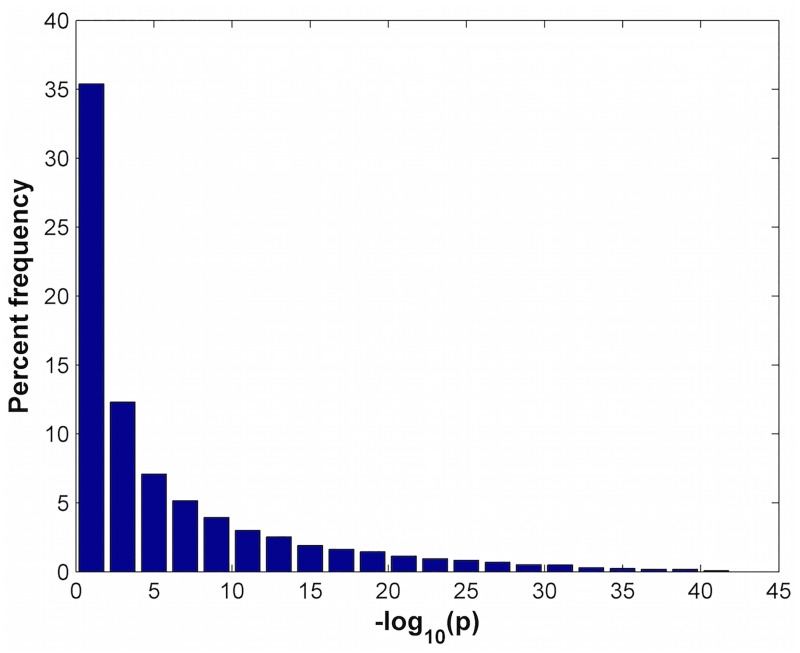
Relative frequency distribution of p-values in GSE13204 when comparing 174 T-ALL with 74 normal patients using the t-test. Note the high percentage of genes with very low p-values.

When analyzing microarray databases from Gene Expression Omnibus Accession numbers GSE13204 and GSE36133, we find that many of the individual genes are differentially expressed at low p-values. Fig 7 shows the percent frequency of 20,705 genes as a function of p-value for GSE13204 when comparing 174 T-ALL patients with 74 normal patients. The p-value of each gene was calculated using the t-test. The first bar plots the percentage of genes whose −*log*_10_(*p*) values range from 0 to 2 (or equivalently whose p-values range from 10^−2^ to 1), the second bar plots the percentage of genes whose −*log*_10_(*p*) values range from 2 to 4 (or equivalently whose p-values range from 10^−2^ to 10^−4^), the third bar plots the percentage of genes whose −*log*_10_(*p*) values range from 4 to 6, etc. Thus, over 64% of the genes in GSE13204 have p-values of 10^−2^ or lower. Not surprisingly, we find that many of the gene sets are also differentially expressed at very low p-values.

In an attempt to isolate a few biological themes among many differentially expressed gene sets, we decide to select the gene sets with the smallest p-values. We acknowledge that this approach will underreport many of the differentially expressed gene sets. However, due to the large number of sets, it would not be useful to report all the differentially expressed gene sets.

Furthermore, databases GSE13204 and GSE36133 require a prohibitively large number of permutations to differentiate the statistical significance of gene sets since the computed p-value of a gene set cannot be smaller than the reciprocal of the number of permutations used. We find that even with a million permutations, a large percentage of gene sets would share the smallest p-value (1 × 10^−6^) available. To overcome this problem, we extrapolate the correlated or permutation based p-value by computing coefficients *m* and *b* using the linear regression curve Eq (5) using 100,000 permutations. Eq (4) then allows us to extrapolate a very small permutation p-value using a regression line constructed with larger p-values.

Tables 8 and 9 show the gene sets (from BioCarta, Kegg, Reactome, and Hallmark) with the highest level of differential expression using datasets GSE13204 and GSE36133 respectively with false discovery rates of 1 × 10^−70^ and 3 × 10^−20^. The subset of GSE13204 we use contains 174 T-ALL patients and 74 normal patients. The subset of GSE36133 we use contains 13 T-ALL patients and 33 AML patients. The t-test is used to compute p-values of individual genes for GSE13204 and the Wilcoxon rank-sum test is used to compute p-values of individual genes for GSE36133. The largest permutation p-value of all the gene sets listed in Table 7 from GSE13204 is 2.6 × 10^−73^ while the largest permutation p-value of the genes sets listed in Table 9 is 1.7 × 10^−22^. We use the modified Fisher’s method with *p*_*min*_ = 1 × 10^−8^ for GSE13204 and *p*_*min*_ = 10^−5^ for GSE36133 along with a regression line to generate the permutation p-value for the gene set. Descriptions of the gene sets from Subramanian [2] are included in the table captions. We note that only large gene sets (sets with greater than 142 genes) are selected since smaller gene sets cannot achieve the very small p-values the larger gene sets can attain.

**Table 8:** Gene sets that are differentially expressed (T-ALL versus Healthy) using Gene Expression Omnibus Accession GSE13204 using a False Discovery Rate of 1 × 10^−70^. Individual genes within each set can be found at software.broadinstitute.org/gsea/msigdb [2]. Individual gene p-values are computed with the t-test. **Description of gene sets in Table 8** from Subramanian et al. [2]. 1. “Genes down-regulated in response to ultraviolet (UV) radiation” 2. “Genes involved in Signalling by NGF” 3. “Cell-matrix adhesions play essential roles in important biological processes including cell motility, cell proliferation, cell differentiation, regulation of gene expression and cell survival. At the cell-extracellular matrix contact points, specialized structures are formed and termed focal adhesions, where bundles of actin filaments are anchored to transmembrane receptors of the integrin family through a multi-molecular complex of junctional plaque proteins.” 4. “Genes down-regulated by KRAS activation” 5. “Regulation of actin cytoskeleton” 6. “Genes up-regulated in response to low oxygen levels (hypoxia)” 7.“Endocytosis is a mechanism for cells to remove ligands, nutrients, and plasma membrane (PM) proteins, and lipids from the cell surface, bringing them into the cell interior.” 8.“Genes encoding components of apical junction complex”.

	GENE SET	DATABASE	Number of genes
1	HALLMARK_UV_RESPONSE_DN	MSigDB Hallmark	144 (142)
2	REACTOME_SIGNALLING_BY_NGF	Reactome	217 (211)
3	KEGG_FOCAL_ADHESION	KEGG pathway	201 (197)
4	HALLMARK_KRAS_SIGNALING_DN	MSigDB Hallmark	200 (199)
5	KEGG_REGULATION_OF_ACTIN_CYTOSKELETON	KEGG pathway	216 (209)
6	HALLMARK_HYPOXIA	MSigDB Hallmark	200 (200)
7	KEGG_ENDOCYTOSIS	KEGG pathway	183 (179)
8	HALLMARK_APICAL_JUNCTION	MSigDB Hallmark	200 (200)

**Table 9:** Gene sets that are differentially expressed (T-ALL versus AML cancer) using Gene Expression Omnibus Accession GSE36133 using a False Discovery Rate of 3 × 10^−20^. Individual genes within each set can be found at software.broadinstitute.org/gsea/msigdb [2]. Individual gene p-values are computed with the Wilcoxon rank-sum test. **Description of gene sets in Table 9** from Subramanian et al. [2]. 1. “Genes encoding cell cycle related targets of E2F transcription factors” 2. “Genes involved in the G2/M checkpoint, as in progression through the cell division cycle” 3.“Genes important for mitotic spindle assembly” 4. “Genes involved in DNA Replication” 5. “Genes involved in Mitotic M-M/G1 phases” 6. “Genes up-regulated during transplant rejection.” 7. “Genes encoding components of the complement system, which is part of the innate immune system” 8. “Genes involved in Signalling by NGF (nerve growth factor)” 9. “Genes up-regulated by STAT5 in response to IL2 (Interleukin 2) stimulation” 10. “Genes regulated by NF-kB in response to TNF (Tumor Necrosis Factor) [GeneID = 7124]” 11. “Genes mediating programmed cell death (apoptosis) by activation of caspases”.

	GENE SET	DATABASE	Number of genes
1	HALLMARK_E2F_TARGETS	MSigDB Hallmark	200 (190)
2	HALLMARK_G2M_CHECKPOINT	MSigDB Hallmark	200 (195)
3	HALLMARK_MITOTIC_SPINDLE	MSigDB Hallmark	200 (198)
4	REACTOME_DNA_REPLICATION	Reactome	192 (178)
5	REACTOME_MITOTIC_M_M_G1_PHASES	Reactome	172 (158)
6	HALLMARK_ALLOGRAFT_REJECTION	MSigDB Hallmark	200 (196)
7	HALLMARK_COMPLEMENT	MSigDB Hallmark	200 (195)
8	REACTOME_SIGNALLING_BY_NGF	REACTOME	217 (211)
9	HALLMARK_IL2_STAT5_SIGNALING	MSigDB Hallmark	200 (194)
10	HALLMARK_TNFA_SIGNALING_VIA_NFKB	MSigDB Hallmark	200 (197)
11	HALLMARK_APOPTOSIS	MSigDB Hallmark	161 (153)

The most highly ranked gene sets in GSE13204 that differentiate T-ALL patients from healthy patients regulate unexpected mechanisms (UV response, hypoxia), signalling mechanisms (nerve growth factor (NGF) and KRAS), cell-matrix adhesions and apical junctions, the cytoskeleton, and endocytosis. RAS signalling and KRAS are identified as mutations in ETP ALL in Zhang et al. [34]. The most highly ranked gene sets in GSE36133 that differentiate T-ALL patients from AML patients regulate transcription factors, mitosis, response to cytokine stimulation, and apoptosis.

While they do not rank highest, all the genes sets listed in Table 7 with GSE46170 are also significant in GSE13204 with gene p-values {1.2 × 10^−12^, 4.1 × 10^−34^, 1.8 × 10^−20^, 2.6 × 10^−22^, 1.2 × 10^−24^, 3.6 × 10^−32^, 3.2 × 10^−12^, 7.3 × 10^−29^, 5.1 × 10^−20^} respectively. Among these gene sets, the p-values of REACTOME_PRE_NOTCH_TRANSCRIPTION_AND_TRANSLATION and REACTOME_SIGNALING_BY_BMP are the smallest, while the p-values of BIOCARTA_P35ALZHEIMERS_PATHWAY and REACTOME_ELEVATION_OF_CYTOSOLIC_CA2_LEVELS are the largest.

## 6 Discussion

Fisher’s method is a self-contained method used to compute the consolidated p-value of a gene set. We show that Fisher’s method has a high level of correlation with many other self-contained methods. We modify Fisher’s method to require the differential expression of multiple individual genes in order to trigger the differential expression of the entire gene set. Dependencies among the gene sets can be computed during the permutation process and displayed using a heat map. Our method is applied to study the differential expression of precompiled gene sets from the MSigDB database. We use microarray databases GSE46170, GSE13204, and GSE36133 from the Gene Expression Omnibus to study the differential expression of gene sets for T-ALL vs Healthy patients and T-ALL vs AML patients and display the results in Tables 7, 8 and 9. We find that we need to extrapolate the permutation p-value for databases (GSE13204 and GSE36133) which contain a large percentage of highly differentially expressed genes.

From our gene set analysis, we are able to identify gene sets associated with Pre-NOTCH transcription and translation as well as genes down-regulated by KRAS activation which have been previously associated with T-ALL. We also identify many gene sets that may not have immediate ties to T-ALL in regards to its genetic signature, and which would require additional scrutiny of its individual genes.

We believe our self-contained method is innovative because: it requires the involvement of multiple individual genes; it is capable of displaying dependencies among genes; and it can compute the permutation p-value of highly differentially expressed gene sets. Future efforts would attempt to put large and small gene sets on equal statistical footing, since large gene sets tend to be selected over small gene sets, when a large portion of gene sets are differentially expressed.
